# Interments in the City and Liberties of Philadelphia, from the 26th of June, to the 3d of July

**Published:** 1841-07-10

**Authors:** 


### HEALTH OF THE CITY.

Interments *in the City and Liberties of Philadelphia, from the* 26*th of June, to the* 3*d of July.*

| Diseases. | Adults. | Children. | Diseases. | Adults. | Children. |
|---|---|---|---|---|---|
| Asphyxia, | 0 | 1 | *Brought forward,* | 31 | 46 |
| Apoplexy, | 3 | 0 | Intemperance, | 2 | 0 |
| Croup, | 0 | 1 | Inanition, | 0 | 2 |
| Congestion of Brain, | 1 | 0 | Infanticide, | 0 | 1 |
| Consumption of the lungs, | 9 | 0 | Marasmus, | 1 | 10 |
| Convulsions, | 1 | 4 | Measles, | 0 | 1 |
| —— Puerperal, | 1 | 0 | Neglect, | 0 | 1 |
| Cachexia, | 0 | 1 | Old age, | 2 | 0 |
| Diarrhœa, | 0 | 6 | Scirrhus of Uterus | 1 | 0 |
| Dropsy, | 0 | 1 | Small pox, | 0 | 5 |
| —— Abdominal, | 2 | 0 | Still-born, | 0 | 7 |
| —— Head, | 0 | 9 | Summer Complaint, | 0 | 17 |
| Disease of Heart, | 2 | 1 | Unknown, | 1 | 1 |
| —— Bowels, | 1 | 0 | Varioloid, | 0 | 1 |
| Drowned, | 2 | 1 | | — | — |
| Dysentery, | 1 | 1 | Total, | 130—38 | 92 |
| Debility, | 0 | 3 | | | |
| Effects of Lightning, | 1 | 0 | | | |
| Fever, | 3 | 0 | | | |
| —— Congestive, | 1 | 0 | | | |
| —— Bilious, | 0 | 1 | | | |
| —— Typhoid, | 1 | 0 | | | |
| —— Inflammatory, | 0 | 1 | | | |
| Hæmorrhage from Bowels, | 1 | 0 | | | |
| Inflammation of the Brain, | 0 | 2 | | | |
| —— Bronchi, | 1 | 2 | | | |
| —— Lungs, | 0 | 6 | | | |
| —— Stomach and Bowels, | 0 | 1 | | | |
| —— Bowels, | 1 | 2 | | | |
| —— Liver, | 0 | 1 | | | |
| | — | — | | | |
| *Carried forward,* | 31 | 46 | | | |

Of the above, there

| | |
|---|---|
| were under 1 year, | 61 |
| From 1 to 2 | 13 |
| 2 to 5 | 11 |
| 5 to 10 | 5 |
| 10 to 15 | 1 |
| 15 to 20 | 1 |
| 20 to 30 | 10 |
| 30 to 40 | 7 |
| 40 to 50 | 8 |
| 50 to 60 | 4 |
| 60 to 70 | 5 |
| 70 to 80 | 2 |
| 80 to 90 | 2 |
| 90 to 100 | 0 |
| Total, | 130 |

Of the above there were 6 from the alms-house, 15 people of colour, and 2 from the country, which are included in the total amount.

